# The Impact of COVID-19 Pandemic and Lockdown Measures on Quality of Life among Italian General Population

**DOI:** 10.3390/jcm10020289

**Published:** 2021-01-14

**Authors:** Maria Stella Epifanio, Federica Andrei, Giacomo Mancini, Francesca Agostini, Marco Andrea Piombo, Vittoria Spicuzza, Martina Riolo, Gioacchino Lavanco, Elena Trombini, Sabina La Grutta

**Affiliations:** 1 Department of Psychology, Educational Science and Human Movement, University of Palermo, 90128 Palermo, Italy; mariastella.epifanio@unipa.it (M.S.E.); vittoriaspicuzza@hotmail.it (V.S.); martina.riolo93@gmail.com (M.R.); gioacchino.lavanco@unipa.it (G.L.); sabina.lagrutta@unipa.it (S.L.G.); 2 Department of Psychology “Renzo Canestrari”, Alma Mater Studiorum, University of Bologna, 40127 Bologna, Italy; federica.andrei2@unibo.it (F.A.); f.agostini@unibo.it (F.A.); marcoandrea.piombo2@unibo.it (M.A.P.); elena.trombini@unibo.it (E.T.); 3 Department of Education Studies “Giovanni Maria Bertin”, Alma Mater Studiorum, University of Bologna, 40126 Bologna, Italy

**Keywords:** COVID-19, Quality of Life, pandemic, lockdown, gender differences, WHOQOL-BREF, health, health psychology, general population, Italy

## Abstract

The COVID-19 pandemic that has hit the world in the year 2020 has put a strain on our ability to cope with events and revolutionized our daily habits. On 9 March, Italy was forced to lockdown to prevent the spread of the infection, with measures including the mandatory closure of schools and nonessential activities, travel restrictions, and the obligation to spend entire weeks in the same physical space. The aim of this study was to assess the impact of the COVID-19 pandemic and lockdown measures on quality of life (QoL) in a large Italian sample, in order to investigate possible differences in QoL levels related to both demographic and pandemic-specific variables. A total of 2251 Italian adults (1665 women, mainly young and middle adults) were recruited via a snowball sampling strategy. Participants were requested to answer to an online survey, which included demographic and COVID-related information items, and the World Health Organization Quality of Life BREF questionnaire (WHOQOL-BREF). The results showed statistically significant differences in QoL depending on a number of variables, including sex, area of residence in Italy, and being diagnosed with a medical/psychiatric condition. To our knowledge, this is the first study to assess QoL during COVID-19 pandemic in Italy, therefore the present findings can offer guidelines regarding which social groups are more vulnerable of a decline in QoL and would benefit of psychological interventions.

## 1. Introduction

During pandemics, the population’s psychological responses to infection play an important role in both the spreading and containment of the disease, influencing the extent to which psychological distress and social disorder occur [1]. This may be partly explained by those emotional states that frequently mark pandemics, such as uncertainty, confusion, and sense of urgency [2]. In the early stages of a pandemic, feelings of uncertainty prevail, due to the fear of becoming infected and not having the right information about the best methods of prevention and management [3,4,5]. Furthermore, pandemics are associated with various psychosocial stressors, including health threats to oneself and loved ones; significant changes in daily routine, such as restriction in the physical activity behavior (PA) [6,7,8]; separation from family and friends; shortages of food and medicine; wage loss; social isolation due to quarantine or other social distancing measures; and school closures [9]. Serious economic difficulties can also occur if a family’s primary wage earner is unable to work due to illness [1].

For these reasons, the effects of the current COVID-19 pandemic would be more pronounced, more widespread, and longer-lasting than the purely somatic effects of infection, with serious impairment on peoples’ actual and perceived quality of life (QoL). The COVID-19 pandemic that has hit the world in the last 12 months has indeed put a strain on our ability to cope with events and revolutionized our daily habits. In Italy, a state of emergency was declared by the Italian government on 31 January 2020 [10], when two Chinese tourists in Rome tested positive for the SARS-CoV-2. The first case in Italy was recorded in February 2020, and the epidemic rapidly spread, reaching 220 infections on 24 February [11]. The government responded by implementing prevention measures and infection control on 11 March, when the number of infections reached 12,462 and the total deaths were 827. Despite the fact that the infection spread differently between the northern and southern regions of Italy, the increasingly restrictive containment measures led to a total lockdown throughout the country (11 March–3 May 2020). Lockdown measures included the mandatory closure of schools and nonessential commercial activities and industries, in addition to travel restrictions both inside and outside the country. After 3 May, the number of infections dropped below 1221 new cases and many restrictions were gradually eased [12]. On 3 June, freedom of movement across regions and European countries was restored and other nonessential activities reopened.

Most of the early studies on the psychological impact of COVID-19, published at the beginning of the pandemic, have compared the current situation with the SARS epidemic in 2003 [13,14,15,16]. These studies highlighted the risk for people with suspected or certain infections to experience uncontrolled fear over a long period, not only in relation to the disease but also to the condition of quarantine. During the previous SARS epidemic, a peak of incidence of many psychiatric disorders, such as depression, anxiety, panic attacks, psychomotorial agitation, and suicide, had been reported. Kwek and colleagues [17] brought out the long-term consequences of the pandemic on health and claimed that SARS impaired significantly both QoL and mental functioning at three months from the acute episode. A small number of additional studies conducted during a previous pandemic also showed the consequences of the pandemic on psychological well-being of infected people, highlighting various factors associated with greater psychological distress, including sociodemographic variables, such as being a woman and middle aged adult or having a lower level of education [3,5]. Moreover, the majority of the studies recently reviewed by Brooks and co-workers [18] reported on the negative psychological effects of quarantine, including symptoms of post-traumatic stress, confusion, and anger. Examples of relevant stressors were a long quarantine period, fear of infection, frustration, boredom, inadequate supplies of personal security systems, inadequate information, financial losses, and social stigma.

This evidence has been further supported by an increasing number of publications on mental health demonstrating higher levels of psychological distress among the population during COVID-19 pandemic [19,20,21,22]. For instance, a large Italian study by Rossi and colleagues [19] showed an increase in anxiety and depressive symptoms for people who had lived four weeks of lockdown, and found 37% of the sample with post-traumatic stress symptoms, whereby female gender and younger age were risk factors for worse mental health.

However, while the attention on the consequences of COVID-19 over mental health has been increasing, there is a limited number of international studies on its effects over QoL. Among already published studies, Pieh and co-workers [23] found an average psychological score of the World Health Organization Quality of Life BREF (WHOQOL-BREF) questionnaire significantly lower compared to a study published in 2015 [24]; the study also reported lower scores for younger adults, women, individuals without work, and those with low income. Horesh, Kapel Lev-Ari, and Hasson-Ohayon [25] also reported higher stress levels and lower QoL for women, younger participants, and for people with pre-existing chronic illness. However, to our knowledge, there have been no studies investigating QoL in Italian populations during the COVID-19 pandemic [23,25,26,27,28].

In addition to sociodemographic variables, it has been suggested that other factors might influence QoL during pandemics, such as the difficulty in accessing healthcare services [26,27] and social isolation [29]. Van Ballegooijen and colleagues [27] described considerable levels of stress, a lower QoL, and concerns about access to healthcare during the first eight weeks of the COVID-19 lockdown in the Netherlands and Belgium. With respect to the difficulty in accessing healthcare, a Chinese study showed that the relevant index of QoL decreased with increasing age, due to the presence of chronic diseases in this segment of the population [26]. Regarding social isolation, a British study reported lower levels of wellbeing and QoL for people who felt more isolated than usual during lockdown, whereas the level of perceived social support showed significant positive correlations with QoL [29]. Another study from a Chinese sample showed relatively lower levels of physical and psychological domains of QoL but, interestingly, not in the social and environmental domains [28].

These studies highlight that the pandemic situation, including the measures put in place to contain it, involves various aspects of life and health. Monitoring the state of health requires the measurement of indicators capable of grasping the many subjective and functional dimensions of well-being and QoL. Particularly, the assessment of QoL is increasingly often considered as an integral part of any intervention that aims to promote health and wellness. QoL is actually viewed as an overall and multidimensional indicator of general wellbeing. Indeed, the WHO defines QoL as “an individual’s perception of their position in life in the context of the culture and value systems in which they live, and in relation to their goals, expectations, standards and concern” (p. 1405) [30]. In measuring QoL, the WHOQOL group takes the subjective dimension strongly into account [31]. The ability to feel a certain well-being, regardless of living conditions, is a subjective variable directly related to other dimensions: genetic variables, personality, and life events. It is a set of factors dynamically interacting with each other in a different way through the life span and across different cultures. QoL is not a simple and linear entity, it is indeed a complex, multidimensional construct that, according to the WHO, includes six domains: physical, psychological, social, level of independence, environment, and spirituality/religions/personal beliefs.

The present study aimed to explore the impact that both the COVID-19 emergency and the resulting restrictive measures had on the perception of QoL among Italian general adult population. Additionally, this study aimed to investigate possible differences in QoL depending on sociodemographic variables, such as sex, age, marital status, occupational status, level of education, and area of residence in Italy, as well as specific factors related to the COVID-19 outbreak (e.g., changes in employment status and location, family members or friends infected with Sars-Cov-2, adherence to control and precautions measures, household size during COVID-19 outbreak). Particular reference will be given to the physical, psychological, social, and environmental domains of QoL as measured by the WHOQOL-BREF.

## 2. Materials and Methods

### 2.1. Procedure

An online cross-sectional survey was performed with Qualtrics^®^ (Qualtrics, Provo, UT, USA) Survey Platform. Such a data collection strategy was chosen as it allowed us to reach as many voluntary participants as possible in a phase of forced social distancing. The survey started after 7 weeks of quarantine in Italy (25 April 2020) and was performed for about 6 weeks, until the end of lockdown measures (2 June 2020). This measurement point was selected because significant changes in individuals’ QoL need some time to be perceived by the person. Moreover, this timeframe potentially allowed the population to adjust to the new situation. The sample was recruited via a snowball sampling strategy. A link to Qualtrics questionnaires were sent via e-mail, social networks (Facebook and WhatsApp), and official working platforms (website of the University of Palermo, Italy). The link was shared with personal contacts of the research group members, who in turn passed the survey to their friends and acquaintances. A brief presentation informed the participants about the aims of the study and electronic informed consent, assuring maximum confidentiality in the handling and analysis of the responses, was requested from each participant before starting the investigation. The survey took approximately 30 min to complete. Participation was voluntary and free of charge. To guarantee anonymity, no personal data, which could allow the identification of participants, were collected. Participants could withdraw from the study at any time without providing any justification, and the data were not saved. Only the questionnaire data with a complete set of answers by respondents were considered. The study was conducted in accordance with the Declaration of Helsinki and was approved by the Bioethics Committee of the University of Palermo (n. 4/2020).

### 2.2. Participants

Italian individuals over 18 years of age who were living in Italy at the time of quarantine were eligible for participation to data collection. The recruited sample size through the online survey included 2332 Italian adults, with an attrition rate of approximately 20%. Of 2332 who completed the survey 71 respondents were excluded because of missing demographic data, while a further 10 participants were excluded as they were residents outside Italy at the time of data collection. Our final sample comprised 2251 respondents. Demographic characteristics of the study sample are presented in Table 1.

### 2.3. Measures

#### 2.3.1. Demographic and COVID-Related Information Questionnaire

An ad hoc questionnaire was created to collect demographic data (such as sex, age, marital status, education level, occupational status, region of residence in Italy, and presence of medical and psychiatric diagnosis) and COVID-related information (i.e., changes in employment status and location, number of people residing with the respondent during quarantine, adherence to control measures, knowing someone who tested positive for COVID-19).

#### 2.3.2. World Health Organization Quality of Life BREF Assessment Instrument (WHOQOL-BREF)

The Italian version of the WHOQOL-BREF was used to assess QoL [32,33]. The WHOQOL-BREF is a short version of the WHOQOL-100, developed by the WHO for use in situations in which time is restricted and respondent burden must be minimized, such as in epidemiological surveys. It is a 26 items self-rating questionnaire, and a person-centered instrument, giving scores to overall QoL and its four dimensions: physical health (e.g., sleep quality, energy and tiredness), psychological health (e.g., positive emotion, self-esteem, personal beliefs), social relationships (e.g., social support and sexual activity), and environment (e.g., climate, transportation, and healthcare assistance). Items ask respondents to rate their QoL during the last two weeks and each of them are rated on a 5-point Likert scale. Similarly to the Italian validation study and to the original version of the questionnaire [29,30], internal consistencies for the WHOQOL-BREF were satisfactory, with Cronbach’s alpha values ranging from 0.57 for social relationships and 0.79 for physical health. Reliability for the global score of the WHOQOL-BREF was good (Cronbach’s alpha = 0.88).

### 2.4. Statistical Analyses

Descriptive statistics and frequency analysis were used to investigate demographic characteristics and COVID-related information. Comparisons on these variables by sex (men vs. women) and age range (young, middle, and older adults) were performed using Pearson’s χ^2^ test and Student’s *t* test for independent samples for nominal and continuous demographic variables, respectively.

Analysis of variance (ANOVA) was used to analyze the difference in respondents’ levels of QoL at the global score of the WHOQOL-BREF, while multivariate analysis of variance (MANOVA) was employed to analyze the differences in levels of QoL at domain scores of the WHOQOL-BREF. Statistical analyses were performed using SPSS (version 25) for Windows [34]. In all statistical tests, a *p* value of less than 0.05 was considered significant.

## 3. Results

### 3.1. Demographic Characteristics

As Table 1 shows, the final sample comprised 2251 participants (74% females) collected mainly from the north (41.3%) and south (53.8%) regions of Italy. Respondents were mostly young (age 18–34) and middle (age 35–64) aged adults (41.7% and 52.2% of the entire sample, respectively), while the group of older adults (age 65 and older) was smaller (6.1% of the total sample). Most of them had a university degree (41.2%) or a high school diploma (36.7%), were employed (62.3%), and either single (46.7%) or married (43.5%). With respect to university students (20.2% of the sample), they were enrolled in either social sciences and humanities (53.2%), biotechnical sciences (29.3%), and medical (14.2%) study programs, while a few students did not report their major (3.3%).

With regards to comparisons between men and women in demographic variables, we found statistically significant sex differences in employment status (χ^2^ = 21.25, *p* < 0.001), level of education (χ^2^ = 23.34, *p* = 0.001), and age range (χ^2^ = 7.59, *p* = 0.022). Particularly, women were less often employed than men, so much so that 80% of the unemployed respondents were women, although with higher levels of education (see Table 1). In fact, women reported more often than men to have a university degree (43.4% vs. 36.0% for women and men, respectively) or a postgraduate title (such as PhD; 14.8% vs. 12.3% for women and men, respectively). Moreover, with regards to age distribution, female respondents were mainly from the group of middle adults, while fewer of them fell into the older adults group compared to men.

Table 1 reports that 136 respondents (6.1%) had a psychiatric diagnosis at the time of data collection, with the highest prevalence in women compared to men (χ^2^ = 6.25, *p* = 0.012). Within this group, 47.1% individuals have been diagnosed with anxiety disorders, 41.2% with mood disorders, while the remaining 11.7% with other conditions (e.g., eating and personality disorders).

Yet, 394 participants (17.5%) reported to be in treatment for a medical condition, mainly for circulatory system diseases (24.1%), such as hypertension and heart failure, and endocrine system diseases (19%), such as diabetes and hypothyroidism. No significant differences in the distribution between men and women were detected (χ^2^ = 1.38, *p* = 0.239).

### 3.2. COVID-Related Information

Table 2 shows the results obtained from epidemic-related information. Most participants had their job/study activity moved at home (50.9%), didn’t have any family members or friends diagnosed with COVID-19 (93.6%), were always adherent to control and precautions measures against COVID-19 (62.9%), and had a household size of mainly three to four persons (55.3%).

Concerning sex differences among these variables, we found a significantly different distribution of answers between men and women in the adherence to control and precautions measures against COVID-19 (χ^2^ = 10.28, *p* = 0.006). Particularly, most women (64.4%) reported to be more inclined to always adhere to control and precautions measures against COVID-19, rather than often or not that much, compared to men (58.8%). We did not find any significant sex difference with regards to the distribution of changes in job/study activity (χ^2^ = 5.12, *p* = 0.163), presence of family members or friends infected by COVID-19 (χ^2^ = 1.25, *p* = 0.263), and household size during the outbreak of the disease (χ^2^ = 6.28, *p* = 0.099).

With respect to age range differences, we found a significantly different distribution of answers in the variables changes in job/study activity (χ^2^ = 74.92, *p* < 0.001) and household size during the outbreak of the disease (χ^2^ = 80.45, *p* < 0.001). Particularly, young (50.4%) and middle (53.9%) adults reported to have mainly their job activity moved at home, as well as a household size of three to four persons during lockdown (58.7% and 55.0% for young and middle adults, respectively), compared to older adults who reported no changes in job or job moved at home to the same extent (29% for both), and a house composition of mainly two persons (44.2%). No significant age differences in the adherence to control and precautions measures against COVID-19 (χ^2^ = 1.57, *p* = 0.815), nor in the presence of family members or friends infected by COVID-19 (χ^2^ = 3.62, *p* = 0.163), were detected.

### 3.3. Quality of Life during the Outbreak of COVID-19

Table 3 presents means and standard deviations for WHOQOL-BREF global and domain scores. The overall average score at the WHOQOL-BREF for our sample was 54.48 (*SD* = 7.77). Analyses performed on the single items, showed that the item with the lowest scores was 14 (about the use of spare time), given that 932 (41.4%) participants reported to have little or no time for leisure at the time of data collection; said item refers to the domain environment of the WHOQOL-BREF. Regarding the other three domains of the WHOQOL, items with lowest scores were: item 15 for the physical domain, as 1019 (45.3%) participants reported little or no possibility to do physical activity; item 5 for the psychological domain, with 712 (31.6%) respondents reporting that they were not enjoying their lives at the time of data collection; and item 21 for social relationships, as 843 (37.4%) respondents reported that they were little or not at all satisfied with their sexual life.

#### 3.3.1. Differences in Sex and Age Range

Results of ANOVA analyses showed that WHOQOL global scores differed between male and female participants (*F* (1, 2250) = 9.34, *p* = 0.002), with women reaching lower scores compared to men. No significant differences were found for age range (*F* (2, 2250) = 1.91, *p* = 0.148). About the factor scores of the WHOQOL, two separate MANOVAs were run by taking into account sex and age range as the only between-subject factor. The model where sex was considered showed a significant main effect for this variable (*F* (1, 2250) = 13.51, *p* < 0.001); between-subject tests showed significant differences between men and women in the areas of physical (*F* (1, 2250) = 17.58, *p* < 0.001), psychological (*F* (1, 2250) = 25.85, *p* < 0.001), and environmental (*F* (1, 2250) = 7.00, *p* = 0.008) domains. As can be seen in Table 3, women reported overall worse psychological, physical, and environmental QoL during the pandemic compared to men.

Age range also resulted in a significant between-subject factor for the detection of differences across WHOQOL-BREF domains (*F* (1, 2250) = 11.93, *p* < 0.001). About this, results showed significant differences among groups in the psychological (*F* (2, 2251) = 11.69, *p* < 0.001) and environmental (*F* (2, 2251) = 11.96, *p* < 0.001) domains. Particularly, young adults reported the lowest levels of psychological QoL, which were significantly lower compared to both middle (*p* < 0.001) and older (*p* = 0.019) adults, as attested by Bonferroni’s post hoc comparisons. As shown by Table 3, middle adults had the lowest scores at the environment domain compared to both young (*p* < 0.001) and older (*p* = 0.005) adults. No significant differences emerged in both physical (*F* (2, 2251) = 0.39, *p* = 0.675) and social relationship (*F* (2, 2251) = 1.82, *p* = 0.161) domains.

#### 3.3.2. Differences in Demographic and COVID-Related Variables

The effects of 10 further relevant variables (i.e., area of residence in Italy, level of education, marital status, employment status, currently diagnosed with psychiatric condition, currently diagnosed with medical condition, changes in employment status and location, family member or friend infected with Sars-Cov-2, adherence to the precautions and control measures, household size during COVID outbreak) were tested over WHOQOL global and domain scores. In light of the results on sex and age range, sex was controlled in all additional ANOVAs, while both sex and age in all MANOVA models. Table 4 presents means, standard deviations and statistics of ANOVA and MANOVA analyses. Overall, no interaction term was significant, therefore statistics were not reported within the Table. As reported by Table 4, results show that seven out of ten variables significantly differed in WHOQOL global score (global level of QoL), while five other WHOQOL factor scores did not (physical, psychological, environmental health, and social relationships; *p* < 0.05). Overall, three variables, namely marital status, family member or friend infected with Sars-Cov-2, and household size during COVID outbreak, had no significant effect over both global and factor scores of the WHOQOL (*p*s = n.s.).

Regarding WHOQOL global score, results from Table 4 show that individuals with the poorest QoL during the outbreak of the disease (as their global score of the WHOQOL was significantly lower compared to the other groups) had the following characteristics: lived in the South of Italy, had lower education levels (secondary or high school diploma), were unemployed or university students, had been diagnosed with psychiatric and medical syndromes, had their job activity suspended, and did not comply with the restriction measures to contrast COVID-19 pandemic.

With respect to the factor scores of the WHOQOL, significant effects were found for the following variables: area of residence in Italy, level of education, having a diagnosis of a medical condition, changes in employment status and location, and for adherence to precaution measures. None of such effects pertained the dimension of the WHOQOL assessing social relationships (all *p*s = n.s.). When area of residence in Italy was considered, between-subject tests revealed that only the differences pertaining the dimension of environmental health were significant (*F* (2, 2250) = 11.16, *p* < 0.001), with respondents living in the south reporting overall worse conditions of their environment, which were significantly different compared to respondents from the north of Italy (*p* < 0.001).

Between-subject tests for level of education showed that environmental (*F* (3, 2145) = 5.43, *p* = 0.001) and psychological health (*F* (3, 2145) = 3.45, *p* = 0.016) were significantly different across groups. Particularly, Bonferroni post hoc tests showed that individuals with a high school diploma had significantly lower levels of psychological health compared to respondents who had either a university degree (*p* = 0.028) or a postgraduate title (*p* < 0.001). Yet, individuals with a postgraduate title reported the highest scores for environmental health, which were significantly different to that of individuals with a secondary (*p* < 0.001) or high school (*p* < 0.001) diploma, as well as with a university degree (*p* = 0.040).

With respect to medical conditions, between-subject tests showed that physical (*F* (3, 2145) = 8.91, *p* = 0.003), psychological (*F* (3, 2145) = 4.03, *p* = 0.045), and environment (*F* (3, 2145) = 4.90, *p* = 0.027) domains of QoL were significantly lower for those respondents reporting a diagnosis of a medical condition.

Between-subject tests relevant to changes in employment status and location showed significant differences across groups in both physical (*F* (3, 2250) = 5.97, *p* < 0.001) and psychological domains (*F* (3, 2250) = 4.21, *p* = 0.006). Specifically, respondents who were unemployed prior to the COVID-19 outbreak reported worse levels of both physical and psychological health, which were significantly lower compared to individuals who had their job/study activity with no changes (*p* < 0.001 for both physical and psychological domains) or moved to home (*p* = 0.001 and *p* = 0.012 for physical and psychological domains, respectively).

With respect to the variable adherence to control measures, between-subject tests showed that the domain environment (*F* (3, 2145) = 6.15, *p* = 0.002) was significantly different across groups, with individuals who reported lower levels of adherence to control measures having the poorest QoL pertaining to environment, compared to respondents who reported either always or often (both *p*s < 0.001).

## 4. Discussion

The study aimed to assess the impact of the COVID-19 pandemic and lockdown measures on QoL in a large Italian sample. The main objective was to investigate possible differences in QoL levels related to both demographic and pandemic-specific factors, with particular attention to physical, psychological, social, and environmental dimensions of QoL. Our results show a number of significant differences in QoL levels related to several relevant variables.

Although the WHOQOL does not have cut-off scores allowing a precise definition of QoL as “poor” or “good”, and despite the absence of recent data available on Italian QoL assessed with the WHOQOL, already existing literature can be taken into account to make some general considerations. Our results showed that, during the lockdown period, the mean of both the global and dimensions scores of the WHOQOL were lower compared to those obtained by both the Italian validation study of the questionnaire [33] and an international study comparing the main psychometric properties of WHOQOL-BREF among 23 countries [31]. Along this line, it is interesting to note that our results showed a poorer QoL for our sample compared to the data reported by another Italian study, in which the goal was to estimate QoL changes over an 18-month period in an adult population sample after the L’Aquila 2009 earthquake [35]. These results emphasize that the current situation due to the pandemic emergency and the lockdown measures had a severe impact on the QoL of the Italian general population, as confirmed by ISTAT (The Italian National Institute of Statistics) report [36]. It was, and still is, an actual collective trauma. In fact, although only 7.4% of the respondents reported to have a friend or relatives hit by COVID-19, we did not find significant differences in QoL compared to participants who had no friends or relatives infected by the virus. People’s lives during lockdown were affected by an abrupt and sudden change in their habits, a sense of precariousness, the indefiniteness of the future, and a strong worry for their health. All these factors may have affected general QoL levels.

Looking into this even further, we found that the items that overall had the lowest scores were: “To what extent do you have the opportunity for leisure activities?” (item 14—environment dimension), “How well are you able to get around?” (item 15—physical domain), “How much do you enjoy life?” (item 5—psychological domain), and “How satisfied are you with your sex life?” (item 21—social domain). Through these items, it is possible to grasp the considerable impact that the lockdown measures have had on the dimensions of life satisfaction and pleasure, favoring an impairment of the ability to enjoy life. Particular attention should be given to the psychological domain, which seems to indicate a relapse to depressive nuances related to the loss of pleasure for one’s life. Furthermore, it might be that the shelter-in-place order could have led to restrictions in physical activity behavior [6], with a possible significant negative impact on psychological well-being and QoL. In fact, recent literature suggests that daily physical activity helped to offset the psychological burden and negative emotions caused by COVID-19 pandemic [6,7,8]. A possible explanation is that regular exercise is linked to change in hypothalamic–pituitary–adrenal (HPA) axis, with reduced adrenal, autonomic, and psychological responses to a psychosocial stressor [37].

With respect to the influence of demographics on QoL, results showed significant differences between men and women. In line with the literature on QoL, women reported overall worse psychological, physical, and environmental QoL during the pandemic compared to men [31,33]. For instance, Girgus and Yang [38] showed that women’s increased psychological vulnerability might be due to a higher tendency to ruminate and to use internal attribution for negative events. Pineles, Hall, and Rasmusson reported more cognitive symptoms of PTSD, such as self-blame, in women compared to men [39]. It is important to notice that in our sample, 80% of unemployed respondents were women, although with higher levels of education than men. Yet, within the 6.1% of respondents that had a psychiatric diagnosis, the highest prevalence was represented by women. With this regard, epidemiological data have shown that in Italy, despite a higher longevity, women get more illnesses and tend to have a lower quality of physical and psychological health than men [40,41]. According to Bekker [42], gender differences in health-related phenomena can be explained through a holistic approach, in which the relationships between biological sex, gender, and health are various, diverse, operative at many levels, and complex. In fact, this relationship can be moderated by daily life or social circumstances, person-related characteristics, and healthcare factors [42]. With respect to daily life and social circumstances, we can assume that, as a consequence of school closures, during the COVID-19 lockdown Italian women experienced a greater overload in care and work, favoring an organizational family shock [35,43].

With regards to age range differences, young adults (18-34) reported the lowest levels of psychological health, which were significantly lower compared to both middle and older adults. Middle adults had the lowest levels of environment dimension compared to both young and older adults. No significant differences emerged for both physical and social domains. Compared to other age groups and in the context of the pandemic, younger adults represent the most psychologically fragile subjects. Additionally, their age is characterized by important transformations (starting university, graduation, first access to work, precarious work condition, unemployment, sentimental projects), which during the pandemic situation might have exposed them to higher risks for their psychological wellbeing. Students, unemployed young people, or young people in the process of building a family or achieving working objectives have suddenly seen a threat to their projects and prospects for the future (finding a job, getting married). Young adults have certainly experienced more negative emotions and loss of self-confidence, with a possible impact on reasoning ability, learning, memory, and concentration, for example for university performances. In fact, emotional skills are crucial to cognitive processes as they affect cognitive styles, use of learning strategies [44], and, consequently, performance [45].

Other studies conducted during lockdown [19,23,25] showed a lower QoL and high levels of stress, anxiety, and depression in younger adults. Pieh and colleagues [23] reported a clear age-related effect in all tested mental health scales, in which the younger adult groups showed the worst scores, in contrast to a previous study before COVID-19. The authors hypothesized various explanations for these findings, such as more uncertain conditions and financial difficulties that occurred in COVID-19 lockdown. According to Horesh and co-workers [25], instead, older age seemed to act as a protective factor for psychological health and this could be attributed to their richer life experience [46] and a possible reduced fear of illness and death, despite the fact that the elderly are constantly being identified as a high-risk population [26,47,48,49]. Middle adults showed less impact on mental health but greatest dissatisfaction with the availability of financial resources, accessibility and quality of health and social care [26,27], the domestic environment conditions, access to information and sense of safety for their own health regarding to the physical environment, and to the possibility to access to means of transport in safety, compared to younger and older ones.

During lockdown, about 50% of young people and 53% of middle adults underwent changes in work conditions (moved home). This can also explain the dissatisfaction about housing conditions, in which simultaneously parents and children shared the same spaces to carry out their activities, with a probable lack of personal space, but about 18% of middle adults and about 14% of older had to stop their work activities, and this could have led to dissatisfaction with their own financial resources, with these not being considered adequate to meet their needs. In addition, in the first weeks after the declaration of emergency state, mass media were overwhelmed by information, which was not always accurate given the little knowledge on the contagion and the care of COVID-19. People probably felt a sense of uncertainty, confusion, and serious threat for their own physical safety. High intolerance of uncertainty has been found to exacerbate the relation between daily stressors and increased anxiety [50] and, not unexpectedly, increased intolerance of uncertainty as well as the desire to reduce uncertainty was found to predict increased information seeking and monitoring of a situation [51]. Therefore, obtaining information that only provides uncertain estimates related to viral threats may serve to increase perceptions of uncertainty and thus increase anxiety [5].

Our results also showed that individuals who were living in the south of Italy at the moment of the lockdown, had lower education levels (secondary or high school diploma), were unemployed or university students, were diagnosed with psychiatric and medical syndromes, had their job activity suspended, and did not comply with the control measures to contrast COVID-19 pandemic had the poorest QoL during the outbreak of the disease. It is interesting to point out that southern Italy, during the first period of lockdown, was less affected by the epidemic, yet the population showed lower levels of satisfaction with their general state of life. On the one hand, this can be related with structural differences that have always recorded lower QoL levels in the south than in the regions of northern Italy [52], especially with regard to the environment dimension (availability of financial resources, access to healthcare services, housing conditions, quality of public transport). Starting from these structural differences between the north and south of Italy, it is possible to assume that the population of southern Italy has perceived greater concern and distrust in the ability to cope with the pandemic. To support this, Rossi and colleagues [19] showed higher odds of several psychological outcomes, such as anxiety, depression, perceived stress, and insomnia in people who lived in southern Italy.

In regards to the relationship between low education level and low scores in the quality of life measure, it appears that the most compromised dimensions were psychological health and the interaction with the environment. Skevington [53] reported worse QoL levels in people without education, especially in some areas of QoL (lack of positive feelings; inadequate financial resources; little information and skills; few opportunities of recreation and leisure; weak spiritual, religious, and personal beliefs). Vice versa, most highly educated respondents reported a more positive environmental QoL, in terms of financial resources and physical environment, e.g., pollution and access to information and skills [53,54]. It is conceivable that, during lockdown, a lower educational level probably impaired more well-being because it hindered access to nonalienated paid work and economic resources, and may have reduced the sense of control over one’s life, as well as the access to stable social relationships, especially marriage. Then, a lower educational level could increase emotional distress (including depression, anxiety, and anger), physical distress (including aches and pains and malaise), and levels of dissatisfaction.

As to work conditions, individuals who were unemployed prior to the COVID-19 outbreak reported overall worse levels of both physical and psychological QoL, which were significantly lower compared to individuals who had maintained their job/study activity with no changes nor moved to home. These findings are supported by previous studies highlighting a relationship between unemployment and poorer health-related QoL, explained by the economic and social consequences of unemployment [55,56]. Work has a central part in most individuals’ lives. It meets the requirements of both material needs (income security and social protection) and social needs (self-esteem and identity, social interaction, time structure, and feeling of purpose and participation in society) [57], and these requirements are further compromised by limitations about job search activities during lockdown [36].

With reference to persons suffering from medical diseases, they reported lower scores in the physical and psychological domains, but also in the interaction with the environment, probably due to the difficulties of access to healthcare services (e.g., concern about cancelled/postponed care). During the pandemic, Italian hospitals were converted into COVID hospitals, and entire wards and surgeries were closed, making it difficult to access for all those with chronic or acute non-COVID-19 medical conditions. Furthermore, as assumed by Van Ballegoijen and co-workers [27], patients could have been anxious to visit their physician due to fear of infection or to avoid further burdening the healthcare system. This could lead to secondary healthcare problems, such as delay in diagnosis of critical medical conditions and exacerbation of existing health conditions. Horesh and colleagues [25] hypothesized that having a pre-existing medical condition is associated to distress, because COVID-19 is more dangerous for those with existing illness and, for that reason, these patients may have felt more vulnerable.

Most of our participants said they adhered to the government-enacted measures much or very much, and there was a significant difference between women and men in favor of the former. These data are in line with the study of Carlucci, D’Ambrosio, and Balsamo [58], where it was assumed that the increased adherence of women to containment measures can explain sex differences in mortality and vulnerability [59,60] to the COVID-19 disease. In this case, women’s adherence has been a protective factor. As suggested by findings from previous studies regarding age and gender patterns of risk-taking behaviors [61,62], men would be more likely to engage in risk taking behaviors.

Finally, the present results have also highlighted that people who felt a greater dissatisfaction in all areas of QoL, especially the environment dimension, had a lower adherence to containment measures. After all, QoL is given by the interaction between environmental and personal factors, and it is possible that people who have perceived higher dissatisfaction with the availability of financial resources, physical safety, and accessibility and quality of health and social assistance may have had a more passive attitude linked to the sense of helplessness, concerning the real possibility that their personal contribution could contain the spread of contagion. Moreover, feelings of helplessness and passivity in dealing with the threat may result from high perception of risk that can promote the adoption of strategies to minimize infection [63].

## 5. Conclusions

There are limited international studies that have investigated how severe the impact of COVID-19 pandemic is on QoL and to our knowledge there have been no studies on the Italian population [23,25,26,27,28]. We believe that the assessment of QoL represents an important indicator of global health, which allows us to grasp the state of health of a population in a multidimensional way, especially in this particular moment in which all the dimensions of life have been disrupted.

Our study highlights significant differences in QoL and its dimensions (physical, psychological, environmental, and social) depending on a number of variables, including sex, age, status of employment, area of residence in Italy, and being diagnosed with a medical/psychiatric condition during the COVID-19 pandemic and lockdown. Strengths of the present study include the focus on a large Italian representative sample, which could be reached in a relatively short time period since the pandemic situation developed rapidly, and the use of an internationally validated questionnaire. Of course, the present study has some shortcomings, such as gender imbalance, cross-sectional data collection, the lack of information on the population of the central regions of Italy, and no exclusion criteria except minors under the age of 18 and those not living in Italy during COVID-19 lockdown.

We are aware that we have analyzed only some of multiple aspects that influence QoL and many others should be tested and considered in further research, such as the role of physical activity on psychological well-being. However, based on our findings, attention should be given to people showing a combination of risk factors, including younger age, female gender, unemployed status, having a pre-existing illness, and living in the south of Italy, thereby assisting them in coping with the pandemic, especially now that the continued exposure to the epidemic and to the necessary measures to contain it, above all in Italy, could lead to further impairment of the people’s quality of life.

We believe that subjective well-being measures are needed to assess a society’s population and it is important to add them to the health and economic indicators that are now favored by policymakers. Such measures include QoL, which may be conceptualized as a multidimensional construct that is influenced by personal and objective factors, as well as by their interactions. The subjective evaluation that people make about their living conditions, their expectations, and their beliefs, could also play a very important role for the adherence to both contagion containment measures and vaccination.

Actually, health authorities have devoted relatively little attention to the identification and management of psychological and social factors likely to significantly influence a person’s QoL. Our results can offer guidelines regarding which social groups may be at a high risk of decreasing QoL, revealing areas of vulnerability during a pandemic. This line of research is particularly important for the management of public health interventions, especially in regards to the need for an optimal allocation of resources. Findings suggest the following recommendations for future interventions: (1) more attention needs to be paid to vulnerable groups such as the young, women, unemployed, and people living in the south of Italy, implementing psychological interventions for vulnerable individuals who cope with the long-term consequences of this pandemic; (2) accessibility to medical resources and the public health service systems should be further strengthened and improved; (3) comprehensive crisis prevention and psychological intervention are needed to reduce distress and prevent further impairment of QoL.

## Figures and Tables

**Table 1:** Frequencies (%) of the main demographic characteristics for men, women, and global sample.

Sample Characteristics	Men (*n* = 586)	Women (*n* = 1665)	Total (*n* = 2251)	*p* Value
Age range (years)				0.022
18–34	247 (42.2)	691 (41.6)	938 (41.7)	
35–64	290 (49.5)	885 (53.1)	1175 (52.2)	
≥65	49 (8.3)	89 (5.3)	138 (6.1)	
Area of residence in Italy				0.087
North	244 (41.6)	686 (41.2)	930 (41.3)	
Center	19 (3.3)	92 (5.5)	111 (4.9)	
South	323 (55.1)	887 (53.3)	1210 (53.8)	
Level of education ^a^				0.001
Secondary school	37 (6.3)	57 (3.4)	94 (4.2)	
High school	249 (42.5)	578 (34.7)	827 (36.7)	
University	211 (36.0)	722 (43.4)	933 (41.4)	
Post-graduate	72 (12.3)	247 (14.8)	319 (14.2)	
Marital status				0.573
Single	269 (45.9)	783 (47.2)	1052 (46.7)	
Married	259 (44.2)	721 (32.0)	980 (43.5)	
Divorced/separated	49 (8.4)	127 (7.7)	176 (7.9)	
Widowed	9 (1.5)	34 (2.1)	43 (1.9)	
Employment status				0.000
Student	114 (19.4)	341 (20.5)	455 (20.2)	
Employed	389 (66.4)	1013 (60.8)	1402 (62.3)	
Unemployed	38 (6.5)	216 (13.0)	254 (11.3)	
Retired	45 (7.7)	95 (5.7)	140 (6.2)	
Currently diagnosed with psychiatric condition				0.012
Yes	23 (3.9)	113 (6.8)	136 (6.1)	
No	563 (96.1)	1552 (93.2)	2115 (93.9)	
Currently diagnosed with medical condition				0.239
Yes	112 (19.1)	282 (16.9)	394 (17.5)	
No	474 (80.9)	1383 (83.1)	1857 (82.5)	

^a^ Global sample size for this variable was 2173, as 61 women and 17 men did not report data on their education level.

**Table 2:** Frequencies (%) of COVID-related information for men, women, and global sample by age range.

	Men				Women				Global Sample			
Sample Characteristics	Young Adults (*n* = 247)	Middle Adults (*n* = 290)	Older Adults (*n* = 49)	Total (*n* = 586)	Young Adults (*n* = 691)	Middle Adults (*n* = 885)	Older Adults (*n* = 89)	Total (*n* = 1665)	Young Adults (*n* = 938)	Middle Adults (*n* = 1175)	Older Adults (*n* = 138)	Total (*n* = 2251)
Changes in employment status and location												
No changes	62 (25.1)	62 (21.4)	15 (30.6)	139 (23.7)	166 (24.0)	165 (18.6)	25 (28.1)	356 (21.3)	228 (24.3)	227 (19.3)	40 (29.0)	495 (22.0)
Job/study activity moved at home	118 (47.8)	154 (53.1)	16 (32.6)	288 (49.1)	355 (51.4)	478 (54.0)	24 (27.0)	857 (51.5)	473 (50.4)	632 (53.8)	40 (29.0)	1145 (50.9)
Job/study activity suspended	44 (17.8)	51 (17.6)	7 (14.3)	102 (17.4)	110 (15.9)	132 (14.9)	9 (10.1)	251 (15.1)	154 (16.4)	183 (15.6)	16 (11.6)	353 (15.7)
Unemployed prior to COVID-19	23 (9.3)	23 (7.9)	11 (22.5)	57 (9.8)	60 (8.7)	110 (12.5)	31 (34.8)	201 (12.1)	83 (8.9)	133 (11.3)	42 (30.4)	258 (11.4)
Family member or friend infected with Sars-Cov-2												
Yes	13 (5.3)	20 (6.9)	0 (0)	33 (5.6)	44 (6.4)	67 (7.6)	5 (5.6)	116 (7.0)	57 (6.1)	87 (7.4)	5 (3.6)	145 (6.4)
No	234 (94.7)	270 (93.1)	49 (100)	553 (94.4)	647 (93.6)	818 (92.4)	84 (94.4)	1549 (93.0)	881 (93.9)	1088 (92.6)	133 (96.4)	2106 (93.6)
Adherence to the precautions and control measures												
Always	140 (56.7)	170 (58.6)	31 (63.3)	341 (58.2)	445 (64.4)	570 (64.4)	57 (64.1)	1072 (64.4)	585 (62.4)	740 (63.0)	88 (63.8)	1413 (62.9)
Often	94 (38.1)	100 (34.5)	15 (30.6)	209 (35.7)	193 (27.9)	260 (29.4)	23 (25.8)	476 (28.6)	287 (30.6)	360 (30.6)	38 (27.5)	685 (30.2)
Not that much	13 (5.2)	20 (6.9)	3 (6.1)	36 (6.1)	53 (7.7)	55 (6.2)	9 (10.1)	117 (7.0)	66 (7.0)	75 (6.4)	12 (8.7)	153 (6.9)
Household size during COVID-19 outbreak												
1 person	25 (10.0)	42 (14.5)	7 (14.3)	74 (12.6)	38 (5.5)	95 (10.7)	19 (21.3)	152 (9.1)	63 (6.7)	137 (11.7)	26 (18.8)	226 (10.0)
2 persons	52 (21.1)	69 (23.8)	22 (44.9)	143 (24.4)	152 (22.0)	228 (25.8)	39 (43.8)	419 (25.2)	204 (21.8)	297 (25.3)	61 (44.2)	562 (25.0)
3–4 persons	137 (55.5)	155 (53.4)	18 (36.7)	310 (52.9)	414 (60.0)	492 (55.6)	28 (31.5)	934 (56.1)	551 (58.7)	647 (55.0)	46 (33.3)	1244 (55.3)
5 persons or more	33 (13.4)	24 (8.3)	2 (4.1)	59 (10.1)	87 (12.5)	70 (7.9)	3 (3.4)	160 (9.6)	120 (12.8)	94 (8.0)	5 (3.7)	219 (9.7)

**Table 3:** Means ± standard deviations of quality of life indicators for all respondents during the outbreak of COVID-19 (*N* = 2251) by sex and age range.

	Young Adults			Middle Adults			Older Adults			Global Sample		
Variables	Women (*n* = 691)	Men (*n* = 247)	Total (*N* = 938)	Women (*n* = 885)	Men (*n* = 290)	Total (*N* = 1175)	Women (*n* = 89)	Men (*n* = 49)	Total (*N* = 138)	Women (*n* = 1665)	Men (*n* = 586)	Total (*n* = 2251)
WHOQOL												
Total score	54.11 ± 8.01	54.97 ± 8.05	54.34 ± 8.03	54.07 ± 7.56	55.59 ± 7.69	54.44 ± 7.61	54.18 ± 7.74	55.32 ± 7.80	54.48 ± 7.77	54.18 ± 7.75	55.32 ± 7.80	54.48 ± 7.77
Physical health	14.11 ± 2.38	14.55 ± 2.29	14.23 ± 2.36	14.02 ± 2.23	14.54 ± 2.24	14.15 ± 2.24	14.24 ± 2.40	14.32 ± 2.04	14.27 ± 2.27	14.07 ± 2.30	14.53 ± 2.25	14.19 ± 2.29
Psychological health	12.82 ± 2.66	13.36 ± 2.54	12.97 ± 2.66	13.30 ± 2.31	13.98 ± 2.38	13.46 ± 2.34	13.39 ± 2.16	13.93 ± 2.32	13.26 ± 2.49	13.11 ± 2.46	13.71 ± 2.50	13.26 ± 2.49
Environmental health	13.56 ± 2.11	13.92 ± 2.16	13.65 ± 2.13	13.18 ± 2.27	13.43 ± 2.15	13.24 ± 2.24	13.96 ± 1.99	13.68 ± 1.91	13.86 ± 1.96	13.38 ± 2.20	13.66 ± 2.14	13.45 ± 2.19
Social relations	13.61 ± 3.15	13.13 ± 3.18	13.49 ± 3.16	13.57 ± 2.88	13.65 ± 2.85	13.59 ± 2.88	14.23 ± 2.50	13.58 ± 2.33	14.00 ± 2.45	13.63 ± 2.98	13.42 ± 2.96	13.57 ± 2.98

**Table 4:** Means ± standard deviations and statistics of ANOVA and MANOVA analyses pertaining respondents’ quality of life during the outbreak of COVID-19 (***n*** = 2251).

	WHOQOL						
	Total Score		Physical Health	Psychological Health	Environmental Health	Social Relations	
Variables		*F*, *p* Value					*F*, *p* Value
Area of residence in Italy		3.86, 0.021					3.34, 0.001
North	55.11 ± 7.28		14.32 ± 2.19	13.45 ± 2.38	13.75 ± 2.08	13.59 ± 2.80	
Center	55.08 ± 7.96		14.26 ± 2.33	13.48 ± 2.36	13.61 ± 2.11	13.73 ± 3.23	
South	53.94 ± 8.09		14.08 ± 2.36	13.10 ± 2.57	13.21 ± 2.25	13.55 ± 3.08	
Level of education ^a^		4.56, 0.003					3.84, 0.003
Secondary school	54.35 ± 9.13		14.26 ± 2.65	13.51 ± 2.81	12.63 ± 2.29	13.94 ± 3.28	
High school	53.71 ± 8.12		14.05 ± 2.41	12.98 ± 2.55	13.16 ± 2.19	13.51 ± 3.06	
University	54.54 ± 7.50		14.22 ± 2.18	13.32 ± 2.43	13.43 ± 2.05	13.57 ± 2.90	
Post-graduate	55.56 ± 7.25		14.37 ± 2.26	13.67 ± 2.35	13.83 ± 2.02	13.67 ± 2.89	
Marital status		0.235, 0.872					1.38, 0.169
Single	54.42 ± 7.81		14.28 ± 2.30	13.08 ± 2.58	13.64 ± 2.08	13.40 ± 3.05	
Married	54.55 ± 7.80		14.09 ± 2.29	13.40 ± 2.38	13.28 ± 2.56	13.77 ± 2.92	
Divorced/separated	54.22 ± 7.34		14.08 ± 2.24	13.45 ± 2.38	13.22 ± 2.24	13.46 ± 2.82	
Widowed	55.51 ± 8.44		14.51 ± 2.31	13.78 ± 2.56	13.57 ± 2.54	13.64 ± 2.72	
Employment status		4.54, 0.004					1.32, 0.198
Student	53.62 ± 7.91		14.02 ± 2.31	12.71 ± 2.67	13.65 ± 2.07	13.25 ± 3.11	
Employed	55.03 ± 7.53		14.31 ± 2.26	13.53 ± 2.37	13.46 ± 2.20	13.72 ± 2.88	
Unemployed	52.75 ± 8.76		13.79 ± 2.44	12.74 ± 2.71	12.91 ± 2.30	13.32 ± 3.34	
Retired	54.87 ± 7.18		14.19 ± 2.23	13.26 ± 2.49	13.71 ± 2.07	13.63 ± 2.71	
Currently diagnosed with psychiatric condition		6.38, 0.012					2.02, 0.089
Yes	52.33 ± 8.66		13.59 ± 2.74	12.42 ± 2.79	13.48 ± 2.36	12.84 ± 3.17	
No	54.62 ± 7.70		14.23 ± 2.26	13.32 ± 2.46	13.45 ± 2.18	13.62 ± 2.96	
Currently diagnosed with medical condition		4.15, 0.042					2.46, 0.043
Yes	53.54 ± 7.94		13.66 ± 2.52	13.12 ± 2.52	13.21 ± 2.23	13.56 ± 2.79	
No	54.67 ± 7.74		14.30 ± 2.23	13.29 ± 2.48	13.51 ± 2.18	13.57 ± 3.02	
Changes in employment status and location		5.78, 0.001					1.94, 0.025
No changes	55.37 ± 7.83		14.41 ± 2.32	13.52 ± 2.55	13.59 ± 2.21	13.85 ± 2.86	
Job/study activity moved at home	54.64 ± 7.22		14.25 ± 2.15	13.32 ± 2.36	13.59 ± 2.10	13.48 ± 2.88	
Job/study activity suspended	53.88 ± 8.52		14.08 ± 2.47	13.06 ± 2.62	13.15 ± 2.31	13.59 ± 3.24	
Unemployed prior to COVID-19	52.87 ± 8.65		13.66 ± 2.52	12.80 ± 2.64	12.97 ± 2.27	13.44 ± 3.22	
Family member or friend infected with Sars-Cov-2		0.164, 0.686					0.96, 0.430
Yes	54.33 ± 7.48		14.12 ± 2.31	13.14 ± 2.39	13.50 ± 1.92	13.56 ± 2.95	
No	54.49 ± 7.80		14.19 ± 2.29	13.27 ± 2.49	13.45 ± 2.21	13.57 ± 2.98	
Adherence to the precautions and control measures		7.26, 0.001					2.03, 0.039
Always	54.88 ± 7.96		14.21 ± 2.35	13.37 ± 2.53	13.59 ± 2.14	13.71 ± 3.00	
Often	54.18 ± 7.41		14.24 ± 2.18	13.22 ± 2.41	13.37 ± 2.15	13.34 ± 2.97	
Not that much	52.05 ± 7.16		13.73 ± 2.24	12.47 ± 2.27	12.53 ±2.52	13.32 ± 2.70	
Household size during COVID outbreak		1.92, 0.125					0.91, 0.531
1 person	55.43 ± 7.06		13.46 ± 2.10	13.63 ± 2.43	13.80 ± 2.10	13.53 ± 2.59	
2 persons	54.76 ± 7.95		14.19 ± 2.36	13.32 ± 2.43	13.42 ± 2.29	13.82 ± 2.95	
3–4 persons	54.33 ± 7.80		14.17 ± 2.28	13.20 ± 2.51	13.42 ± 2.17	13.53 ± 3.04	
5 persons or more	53.62 ± 7.81		13.97 ± 2.36	13.07 ± 2.55	13.34 ± 2.12	13.24 ± 3.04	

^a^ Global sample size for this variable was 2173, as 61 women and 17 men did not report data on their education level.

## Data Availability

Data available on request due to restrictions (privacy).
